# YPD-SLAM: A Real-Time VSLAM System for Handling Dynamic Indoor Environments

**DOI:** 10.3390/s22218561

**Published:** 2022-11-07

**Authors:** Yi Wang, Haoyu Bu, Xiaolong Zhang, Jia Cheng

**Affiliations:** College of Electrical Engineering, North China University of Science and Technology, Tangshan 063210, China

**Keywords:** dynamic environment, low texture, Yolo-FastestV2, SLAM, positional estimation, target detection, planar

## Abstract

Aiming at the problem that Simultaneous localization and mapping (SLAM) is greatly disturbed by many dynamic elements in the actual environment, this paper proposes a real-time Visual SLAM (VSLAM) algorithm to deal with a dynamic indoor environment. Firstly, a lightweight YoloFastestV2 deep learning model combined with NCNN and Mobile Neural Network (MNN) inference frameworks is used to obtain preliminary semantic information of images. The dynamic feature points are removed according to epipolar constraint and dynamic properties of objects between consecutive frames. Since reducing the number of feature points after rejection affects the pose estimation, this paper innovatively combines Cylinder and Plane Extraction (CAPE) planar detection. We generate planes from depth maps and then introduce planar and in-plane point constraints into the nonlinear optimization of SLAM. Finally, the algorithm is tested on the publicly available TUM (RGB-D) dataset, and the average improvement in localization accuracy over ORB-SLAM2, DS-SLAM, and RDMO-SLAM is about 91.95%, 27.21%, and 30.30% under dynamic sequences, respectively. The single-frame tracking time of the whole system is only 42.68 ms, which is 44.1%, being 14.6–34.33% higher than DS-SLAM, RDMO-SLAM, and RDS-SLAM respectively. The system that we proposed significantly increases processing speed, performs better in real-time, and is easily deployed on various platforms.

## 1. Introduction

Simultaneous localization and mapping (SLAM) systems effectively solve autonomous exploration tasks in unknown environments as a fundamental strategy for developing navigation technologies, for example, in mines, roads, farmlands, underwater, aerial environments, and, in a broad sense, indoor and outdoor scenarios. In these scenarios, RGB-D cameras or LiDAR are often used as the primary sensors to capture the scene [1,2]. The Visual SLAM framework is now relatively mature and consists mainly of front-end feature extraction, back-end state estimation, loopback detection, and map building [3]. Some excellent SLAM algorithms, such as ORB-SLAM2 [4], HECTOR-SLAM [5], LSD-SLAM [6], etc., have been applied in some fields with more excellent results.

However, some problems must be solved; for example, most typical algorithms or datasets are based on static environment assumptions. Due to the substantial texture information of dynamic objects in natural scenes, these algorithms use many dynamic object feature points for pose estimation and 3D mapping, resulting in significant trajectory errors and even tracking state loss. As a result, the system based on pose estimation is disturbed by cumulative errors in long-term mapping and localization, which dramatically limits the application of VSLAM in many practical situations.

For indoor environments with a large number of artificial objects and known structures, there are a large number of low-texture scenes in addition to more vital dynamic targets. Such as floors, walls, table tops, etc. Such scenes are usually not valid when using SLAM algorithms based on point feature extraction and even fail to match feature points within low-texture scenes between adjacent frames. In addition, point errors, especially in large scenes, where measurement noise and data correlation accumulate, are challenging to solve using only points. Of course, as one of the ordinary working scenes for robots, the indoor environment has many other high-level features besides feature points. Such as lines, planes, etc. Using plane-type structural constraints can help achieve planar matching and thus reduce the cumulative error. In addition, advanced features such as lines and planes can be easily extracted in RGB-D cameras, and calculating planes from the depth map can make the results more stable and accurate.

In this paper, improvements are made to address the following issues:

1. Based on the assumption of constant environment, ignoring dynamic features leads to inaccurate pose estimation;
2. Traditional pixel-level semantic segmentation networks are inefficient and difficult to meet real-time operation requirements;
3. Removing dynamic feature points from dynamic targets results in a low number of feature points and inaccurate positional estimation;
4. Low-texture scenes are prone to feature matching failures, but in cases with planar features that can be utilized.

The proposed lightweight, fast target detection network based on Yolo-FastestV2 [7] is combined with CAPE [8] (Cylinder and Plane Extraction) based planar detection to extract planar features. We refer to this system as YPD-SLAM based on the approach adopted (YoloFastestV2-Plane-Dynamic-SLAM).

The main contributions of this paper are as follows:

- A SLAM system is proposed based on ORB-SLAM2 that can work in dynamic and low-texture environments;
- Adding the Yolo-FastestV2 target detection network, the threshold of the sum of distances to the epipolar is determined in combination with epipolar constraint to remove dynamic feature points;
- Planar features are extracted based on CAPE planar detection, using planar high-level features of low-textured scenes, and adding planar and in-plane points constraint, thus reducing mismatching and drift errors in indoor environments;
- Evaluating this system on a publicly available dataset, the speed is greatly improved while maintaining the same accuracy as the state-of-the-art systems.

## 2. Related Work

### 2.1. Plane-Based Approach

Planar SLAM uses planar rather than point features as flags for pose estimation and SLAM optimization. Concha and Civera [9] propose a new initialization framework for planar regions by reconstructing high-gradient image regions as 3D points and low-gradient image regions as planes using Super Pixel segmentation. DPPTAM improves the accuracy and density of semi-dense monocular SLAM. Ma et al. [10] perform direct alignment of keyframes and global planar models in the EM framework and optimized constraints between keyframes and global planar models. Lee et al. [11] iteratively estimate layout planes and points cloud alignment to reduce RGBD map offsets.

Similarly, planes can provide constraints over long distances compared to points in indoor architectural environments [12,13]. A keyframe-based framework is proposed by Hsiao et al. [12] to optimize keyframe poses and landmark planes using incremental smoothing and mapping (iSAM). Zhang et al. [14] use planar edges, generate supposed vertical planes, and add planar perpendicular and parallel constraints. More constraints are added to the nonlinear least squares problem for SLAM to achieve a more stable pose estimation. These methods have achieved good results in exploiting planar structures, but the mapping of dynamic indoor environments is less satisfactory.

### 2.2. Semantic-Based Approach in Dynamic Scene

As research progresses, deep learning approaches start to be introduced into SLAM systems, and some deep learning techniques are used to handle these dynamic elements, such as semantic SLAM. A novel SLAM framework proposed by Brasch et al. [15] with semantic networks that use the extracted semantic information and probabilistic models to reject dynamic outliers. In addition, a new visual ranging framework in [16] that incorporates semantic constraints into the pose and map optimization process to reduce the drift caused by dynamic elements. While these methods can extract all possible movable objects from the scene, they do not take into account the temporality of the actual motion, i.e., the features on the objects still contribute to the accuracy of the pose estimation when they are static. Therefore, to take full advantage of all possible features of the object, Bescós et al. [17] propose the DynaSLAM algorithm, which uses Mask R-CNN [18] and multi-view geometry for dynamic segmentation. Ref. [19] uses SegNet [20] to obtain semantic segmentation and use movement consistency checking to re-detect dynamics. Bescós et al. [21] propose a new feature-based dynamic SLAM algorithm for model-free object perception that is still based on Mask R-CNN to estimate the rigid object motion of a rigid object. Liu and Miura [22] add semantic tracking and semantic-based optimization threads based on ORB-SLAM3 in RDS-SLAM. The algorithm also proposes a key frame selection strategy for semantic segmentation, which significantly improves the tracking performance of the system. In 2021, RDMO-SLAM proposed by Liu and Miura [23] sped up Mask R-CNN segmentation using optical flow prediction semantic labels based on RDS-SLAM while adding constraints on optical flow estimation landmark velocity. Su et al. [24] propose a new real-time visualization SLAM algorithm in the tracking thread, introducing a module for optimizing the homography matrix using semantic information. Combining the semantic information, the optimal single response matrix, and the optical flow mask to reject the dynamic feature points in the SLAM front end.

Although this two-stage detector model based on pixel segmentation has high classification accuracy and a low miss recognition rate, it is slow and cannot meet the requirements of real-time scene detection. In general, semantic-based and planar-based approaches have difficulty balancing localization accuracy and real-time performance.

## 3. Method Overview

### 3.1. Overview of YPD-SLAM System

The YPD-SLAM system is mainly based on ORB-SLAM2 and SP-SLAM [14] improvements. The system consists of three main modules: semantic target detection and dynamic point checking, the tracking module, and the map management module.

**Figure 1:**
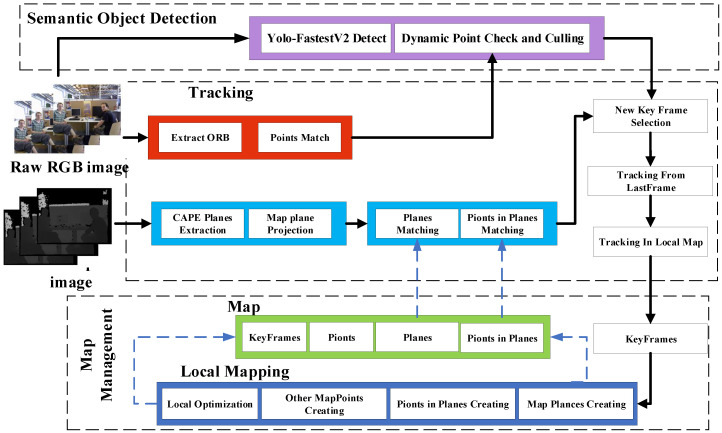
Framework of the YPD-SLAM system.

The block diagram of the proposed system is shown in Figure 1: (1) Semantic target detection and dynamic point checking as in Section 3.2 and Section 3.3 are performed for each frame of the original RGB image, feature extraction, and matching. Extract planes and in-plane points from aligned depth maps as in Section 3.4 and propose new methods as in Section 3.7 for in-plane matching. (2) Section 3.8 estimates the camera’s pose by minimizing the error function posed by the tracked features. (3) In the map management module, update the local mapping consisting of point landmarks, planar landmarks, in-plane points, and keyframes for each newly inserted keyframe. We aim to optimize the poses in low-texture indoor dynamic environments using target detection to remove dynamic effects combined with advanced features of indoor planes.

### 3.2. Semantic Target Detection

The core of the YOLO series of target detection algorithms lies in its small model size and fast computing speed. Unlike the R-CNN series (Fast R-CN [25], Faster R-CNN [26], etc.) algorithms, YOLO is slightly less accurate, but its detection speed is fast. In this paper, we use the fastest single-stage network YOLO-Fastest [27]. The network model is tiny, only 1.3 MB; it reduces power consumption by using one or two processor cores, so it runs very fast, up to 148 frames per second on a single core. It is incredibly versatile, both for multi-platform porting and for easy deployment in PyTorch, Tensorflow, Keras, and Caffe frameworks, i.e., The YOLO-Fastest model size is only 1.3 MB, a 65% reduction in parameters, and a 45% increase in speed compared to the 3.0 MB MobileNet-YOLOv3 [28].

To further satisfy the real-time requirement, we choose YOLO-FastestV2 (the second version of the Yolo-Fastest algorithm). Its model architecture consists of Shufflenet V2 [29] as the backbone and a modified YoloX detection head, where the Anchor matching mechanism is modified from YOLOv5 [30]. Compared to YOLO-Fastest, the accuracy is reduced by only 0.3%, the inference speed is increased by 25%, and the number of parameters is reduced by 25%.

We select the COCO [31] 2017 dataset and train 20 categories. In practice, people, cats, etc. belong to high dynamic categories; chairs, mice, etc. belong to low dynamic categories; tables, sofas, etc. belong to static objects. Therefore, we assume that feature points located on people are most likely to be dynamic points.

### 3.3. Dynamic Point Check

Although DS-SLAM performs motion checking of feature points globally, the system’s real-time operation can be significantly affected by this. In this paper, we use the YOLO FastestV2 target detection network to assign dynamic labels to highly dynamic objects. In contrast, only data with dynamic semantic prior information is processed in the dynamic point rejection algorithm. In our experiments, the small range dynamic feature point detection dramatically improves the operation speed, with a tracking time of only 42.68 ms per frame.

We perform ORB feature extraction and matching and optical flow tracking for the same two frames in the ORB-SLAM2 algorithm; the former takes 135.2 ms, and the latter takes only 6.4 ms. Therefore, the selection of matching point pairs is completed by optical flow tracking for the two adjacent frames of the image after target detection, which takes less time. After optical flow tracking for the points in the prior semantic frame, a threshold is selected to determine whether they are static or dynamic points.

**Figure 2:**
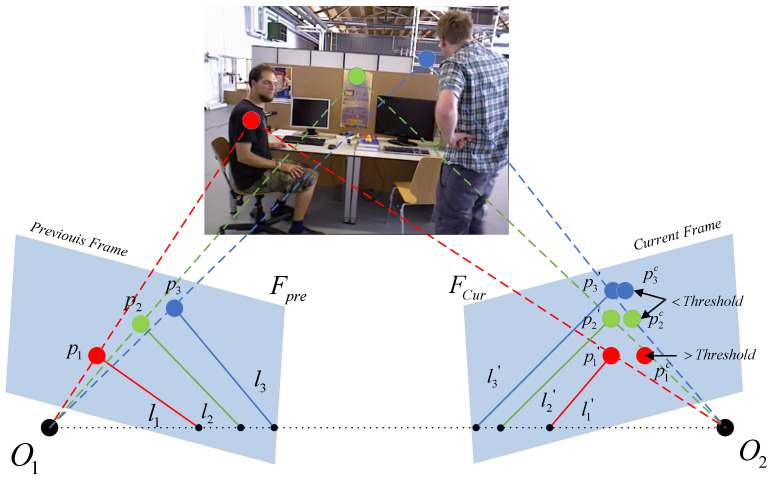
The schematic diagram of dynamic point checking, the left diagram indicates the previous frame and the right diagram indicates the current frame.

As in Figure 2, the geometric relationship between the polar lines of two frames is used to detect dynamic feature points. $O_{1}$ and $O_{2}$ are the camera optical centers of the previous frame $F_{Pre}$ and the current frame $F_{Cur}$, respectively. $p_{1}$, $p_{2}$ and $p_{3}$ denote the partial feature points of $F_{pre}$. $l_{1}$, $l_{2}$, and $l_{3}$ represent the epipolar of $F_{pre}$ with respect to the baseline $O_{1}O_{2}$. According to the correct epipolar constraints, the correct matching points $p_{1}^{\prime },p_{2}^{\prime }$, and $p_{3}^{\prime }$ on $F_{Cur}$ should fall on the corresponding epipolar $l_{1}^{\prime },l_{2}^{\prime }$, and $l_{3}^{\prime }$, respectively. By the epipolar constraint we obtain: $l_{1}^{\prime }=Fp_{1},l_{2}^{\prime }=Fp_{2},l_{3}^{\prime }=Fp_{3}$. In order to meet the real-time requirements as much as possible, we set up only three optical flow pyramids for optical flow tracking to calculate the base matrix *F*. However, the actual matching points of the current frame are often distributed as $p_{1}^{c},p_{2}^{c}$, and $p_{3}^{c}$ due to noise and dynamic points. If the distance from these points (e.g., $p_{2}^{c},p_{3}^{c}$ ) to the corresponding poles is less than a threshold, then they are identified as static points; if they exceed a certain threshold, they do not satisfy the epipolar constraint such as $p_{1}^{c}$ and are recognized as dynamic points. We define the calculation of the distances as follows:

(1) $$D\left(p^{\prime },l^{\prime }\right)=\frac{p^{cT}Fp}{\sqrt{{\left(Fp\right)}_{x}^{2}+{\left(Fp\right)}_{y}^{2}}}$$

where $D\left(p,l^{\prime }\right)$ denotes the distance from point $p^{c}$ to the epipolar $l^{\prime }=Fp$ in the current frame and ${\left(Fp\right)}_{x},{\left(Fp\right)}_{y}$ denotes the non-constant coefficients of $l^{\prime }$.

The specific algorithm flow is shown in Algorithm 1.
**Algorithm 1** Dynamic Points Detection Algorithm**Input:**$F_{pre}$; $F_{cur}$; *P*; *B*;**Output:***S*;1:$P^{c}$ = $CalcOpticalFlowPyrLK(F_{pre},F_{cur},P)$2:*F* = $FindFundamentalMatrix(P,P^{c})$3:**for** each matched pairs $p,p^{c}$**in**$P,P^{c}$ **do**4:    **if** $p,p^{c}$**in**$B_{dynamic}$ **then**5:        L=FindEpipolarLine(p,F)6:        $D=CalcDistanceFromEpipolarLine(p^{c},L)$7:        **if** D>ϵ **then**8:           **continue**;9:        **end if**10:      **continue**;11:    **end if**12:    **Append** $p_{2}$**to***S*13:**end for**

Here, $F_{\mathrm{pre}\;},F_{cur},P$, and *B* denote the previous frame, the current frame, the previous frame feature points, and the frame with semantic prior, respectively; *S* denotes the point set after removing dynamic feature points, and $P^{c}$ denotes the feature points tracked by the current frame optical flow.

### 3.4. Planar Extraction

In this work, for indoor low-texture environments with a large number of artificial structures, we carry out plane detection to extract plane features according to [8] and obtain plane masks, plane cell pixels, and corresponding point cloud planes for feature point optimization and back-end mapping.

**Figure 3:**
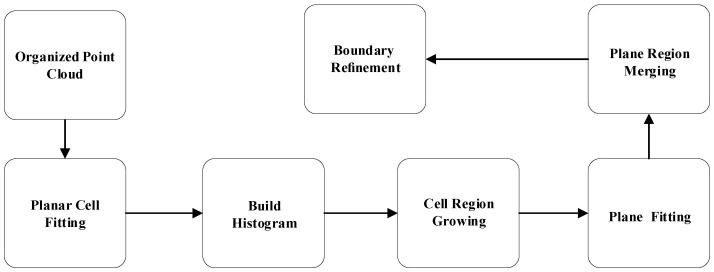
Main flow of planar segmentation [8].

The plane extraction algorithm is divided into five main parts: plane cell fitting, normal histogram, cell-by-cell area growth, plane fitting, and model area refinement, respectively. The planar extraction flowchart is shown in Figure 3. First, the planes are decomposed into pixel blocks and cells at a specified grid resolution for processing. The area growth is performed on these planar cells to find smooth surfaces by creating a normal histogram of the cells (color-coded in the figure) based on a priori information to obtain seed information. In addition, if the planes have similar model parameters and connected cells, they can be merged later. Finally, the boundaries of the regions are refined pixel by pixel within the required cells by morphological operations, and finally, the refined segments are superimposed on the respective RGB images.

The planar segmentation shown in Figure 3 is too coarse and thus requires edge optimization. Traditional methods such as PEAC [32] use very time-consuming pixel-level region growth and do not guarantee accurate results, conflicting with real-time purposes. Therefore, this paper uses 3 × 3 structural elements to perform morphological corrosion operations on the boundary cells, where the corrosion operations are performed using the less influential 4 neighboring domains. Then a morphological expansion operation is performed on the original region using a 3 × 3 kernel with 8 neighboring domains to extend the original region as much as possible. The cells between the expanded and corrupted regions are then marked as white. We calculate the distance between the segmented model and each point within these cells. If the square of this distance is less than k times the segmented model (9 in this paper) and is the minimum distance between any models sharing the refinement cells. Then the cell is assigned to the segmented model.

### 3.5. Point and Plane Representation

The RGB-D camera can capture RGB images as well as aligned depth images. In the depth image, each pixel is related to the distance between the image plane and the corresponding object in the RGB image. Based on the pinhole model in the paper [33], we used the camera model to recover the structure as in Equation (2). For the optimized segmentation plane, each cell’s center of mass is taken out for inverse projection to generate a 3D point cloud.

(2) $$P^{c}=\left(\begin{array}{c} x^{c}\\ y^{c}\\ z^{c} \end{array}\right)=d\left(u^{c}\right)\left(\begin{array}{ccc} f_{x}^{-1} & 0 & -c_{x}f_{x}^{-1}\\ 0 & f_{y}^{-1} & -c_{y}f_{y}^{-1}\\ 0 & 0 & 1 \end{array}\right)\left(\begin{array}{c} u^{c}\\ v^{c}\\ 1 \end{array}\right)\in R^{3}$$

The flush coordinates of normalized depth information are used under the camera coordinate system, $f_{x}$ and $f_{y}$ are the focal lengths of the camera *x* and *y* axes, and ${\left(c_{x},c_{y}\right)}^{T}$ is the camera center coordinate. Here, $u^{c}={\left(u^{c},v^{c}\right)}^{⊤}$ denotes the 2D coordinates of the optimized center-of-mass pixel point in the depth map, and c represents the current frame. For aligned color and depth maps, $u^{c}$ also similarly represents the pixel point coordinates of the color map. $d\left(u^{c}\right)$ represents the depth value corresponding to the depth map point $u^{c}$. The 3D point corresponding to the pixel point of the current frame is denoted as $P^{c}={\left(x^{c},y^{c},z^{c}\right)}^{T}$.

Among the many planar representations, the commonly used planar representation as $\pi ={\left(\pi_{1},\pi_{2},\pi_{3},\pi_{4}\right)}^{⊤}\in P^{3}$. Another Hesse form parametric plane representation as $\pi ={\left(n^{⊤},d\right)}^{⊤}$. Where the normal vector $n={\left(n_{x},n_{y},n_{z}\right)}^{⊤}=\frac{{\left(\pi_{1},\pi_{2},\pi_{3}\right)}^{⊤}}{\sqrt{\pi_{1}^{2}+\pi_{2}^{2}+\pi_{3}^{2}}}$, the plane’s distance from the origin of the current coordinate system $d=\frac{-\pi_{4}}{\sqrt{\pi_{1}^{2}+\pi_{2}^{2}+\pi_{3}^{2}}}$ is represented, and a point P located in plane π satisfies:

(3) $$n^{⊤}P+d=0\;$$

### 3.6. Planar Minimum Representation for Optimization

It is known that the three-dimensional plane has only three degrees of freedom, while the Hesse form of the plane representation has four degrees of freedom. Thus, over-parameterization can lead to singularities in the Hessian matrix computed during Gauss-Newton optimization. The quaternion can solve this problem very well. We project the planar representation onto the tangent space $S^{3}$ and use the quaternion method to optimize the plane.

Specifically, the first three elements of the parametrized plane $\pi^{\prime }$ consist of the normal vector n of the plane, and the fourth element *d* represents a scalar related to the plane’s distance to the origin. Thus, we can obtain the normalized plane $\pi^{\prime }$ denoted by

(4) $$\pi^{\prime }=\frac{\pi }{∥\pi ∥}={\left(\frac{n^{⊤},-d}{∥n^{⊤},d∥}\right)}^{⊤}=\frac{1}{\sqrt{n_{x}^{2}+n_{y}^{2}+n_{z}^{2}+d^{2}}} [\begin{array}{c} n\\ -d \end{array}]\in S^{3}\;$$

Similarly, a tangent space $S^{3}$ can also be represented by a quaternion q. According to the paper [34], here we normalize q to the unit quaternion $q^{\prime }$ used to represent and optimize the rotation, and the first three elements of $q^{\prime }$ denote the rotation vector through the angle θ rotating around the vector $q_{v}$, and the fourth element is the scalar $q_{w}$.

(5) $$q^{\prime }=\left(\begin{array}{c} q_{0}\\ q_{1}\\ q_{2}\\ q_{3} \end{array}\right)=\left(\begin{array}{c} v\sin\left(\frac{\theta }{2}\right)\\ \cos\left(\frac{\theta }{2}\right) \end{array}\right)=\left(\begin{array}{c} q_{v}\\ q_{w} \end{array}\right)\in S^{3},∥q∥=1\;$$

After normalization, the two are located in the same space. Similar to the rotation matrix’s parametric update, we need to update the quaternion parametric update corresponding to the Lie algebra. The exponential mapping allows updating the existing rotation matrix R(q) with an increment $\omega \in R^{3}$. Therefore, the quaternion incremental update is denoted as $q^{s+1}=\exp\left(\omega \right)q^{s}$, where one parametric update is denoted by $q^{s}\to q^{s+1}$. According to the paper [35], using Grassia derives its exponential mapping from $R^{3}$ to $S^{3}$ for optimization:

(6) $$\exp\left(\omega \right)=\left(\begin{array}{c} \left.\frac{1}{2}\mathrm{sinc}\left(\frac{1}{2}∥\omega ∥\right)\omega \right)\\ \cos\left(\frac{1}{2}∥\omega ∥\right) \end{array}\right)\;$$

Conversely, the inverse mapping from $S^{3}$ to $R^{3}$ can be expressed as a three-dimensional rotation vector:

(7) $$\omega =\log\left(q^{\prime }\right)=\frac{2\cos^{-1}\left(q_{w}\right)}{∥q_{v}∥}q_{v}\;$$

So under the same tangent space, there are

(8) $$\log\left(\pi^{\prime }\right)=\frac{2\cos^{-1}\left(\frac{-d}{\sqrt{n_{x}^{2}+n_{y}^{2}+n_{z}^{2}+d^{2}}}\right)}{\sqrt{n_{x}^{2}+n_{y}^{2}+n_{z}^{2}+d^{2}}}n=2\cos^{-1}\left(\frac{-d}{∥n^{⊤},d∥}\right)\frac{n}{∥n^{⊤},d∥}\;$$

The distance $\log\left(\pi_{1}^{-1}⊗\pi_{2}\right)$ between two planes $\pi_{1}$ and $\pi_{2}$ can be solved by tangent space, where ⊗ denotes quaternion multiplication.

### 3.7. Plane and In-Plane Points Match

#### 3.7.1. Plane Match

To get aligned planes from the depth map, we first need to determine whether the planes create a new map plane in the global map or associate the plane with a plane that already exists on the global map. For point features, we use the ORB descriptor to do an initial point match between the current and previous frames. This mapping relationship is then used to project feature points from the previous image frame to the current frame, using a minimization reprojection error to remove some matching outliers. Finally, the optimized matching after error removal.

The set of point pairs is defined as $P^{c,\;L}$, where *c* denotes the 3D feature point of the current frame corresponding to the coordinate system, and *L* denotes the 3D coordinate corresponding to the feature point as a landmark in the local map.

However, there is no corresponding plane descriptor for plane matching, so a novel plane matching method needs to be proposed: for indoor structural environments, planes mostly appear as parallel or perpendicular features, so we look for plane landmarks in the global map that have an intersection relationship with the current frame plane. For the global map, there are roughly two reasons for plane intersection, one is that the same plane produces an intersection with a slight angle due to noise error, and the other is that two different planes are perpendicular in the actual environment so that the intersection angle between perpendicular planes is relatively large. Based on the above analysis, the conditions for the intersection of planes are first defined as follows.

(9) $$S=|{\left(n_{i}^{L}\right)}^{T}P_{ct}+d_{i}^{L}|$$

For planar landmark $\pi_{i}^{L}={\left({\left(n_{i}^{L}\right)}^{T},d_{i}^{L}\right)}^{⊤}$, in order to meet the real-time operation requirements, the 3D planar point centroid $P_{ct}=\frac{1}{n}\left(\sum_{k=0}^{n}x_{k},\sum_{k=0}^{n}y_{k},\sum_{k=0}^{n}z_{k}\right)$ of the current frame $\pi_{j}^{c}$ is used as the calculation distance condition, and *S* represents the distance between the planar centroid of the current frame and the planar landmark in the global map. If *S* is smaller than the distance threshold 0.01 (adjusted several times during the experiment), then the plane of the current frame is considered to intersect with the plane landmark $\pi_{i}^{L}$ in the global map. Next, we only need to exclude the perpendicular cases in the intersecting planes and first calculate the angle $\cos\langle \pi_{i}^{L},\pi_{j}^{c}\rangle$ between the plane landmark $\pi_{i}^{L}$ and the current frame plane $\pi_{j}^{c}$. If $|\cos\langle \pi_{i}^{L},\pi_{j}^{c}\rangle |$ is greater than the threshold $0.9986\left(\approx \cos\left(3^{\circ }\right)\right)$ (adjusted several times in the experiment), we judge that the two planes have finished matching and update the map plane information in the global map.Finally, the set of plane pairs that complete plane matching is noted as $\Pi^{c,L}$.

#### 3.7.2. In-Plane Points Match

If the two planes finish matching, then the points within the planes are matched using Iterative Closest Point (ICP) [36] method. Two problems were encountered in the process of using ICP. Problem 1: Although the number of in-plane points is reduced by the a priori information of plane matching, its overall number is still relatively large. Problem 2: The traditional ICP algorithm requires an iterative initial value, and if the initial value is not selected appropriately, it will have an important impact on the alignment results. In serious cases, it will make the algorithm fall into local optimum, so that the iteration cannot get the correct alignment result.

Therefore, this paper adopts a uniform sampling-based method to reduce the selection of point sets and a KD-tree [37] based method to speed up the nearest point search efficiency. Based on the uniform sampling method, the number of point sets used for ICP is further reduced by collecting every 5 points at intervals in this paper. The KD-tree method is an extension of binary tree in multidimensional space, which is a proposed lookup method for indexing spatial points or multi-attribute data with an average lookup length of $1+4log\left(n\right)$. Point cloud data is a collection of points in space, and each point contains three-dimensional coordinate information, and since point cloud data is irregularly distributed, the KD-tree method is a suitable indexing method for managing point cloud data.

Firstly, the two sets of 3D point clouds *P’* and *P* are preprocessed with data to remove the points with noise. From the above, we calculate the center-of-mass Pct’, and Pct of the two matched 3D planar point clouds, and then calculate the decentered coordinates of each point as follows:

(10) $$\left\{\begin{array}{c} P^{\prime }=\left\{p_{i}^{\prime }-p_{ct_{i}}{}^{\prime }\right\}\\ P=\left\{p_{i}-p_{ct_{i}}\right\} \end{array}\right.$$

Then the in-plane point Euclidean transformation relation can be described as $\forall_{i}P_{i}=R{P_{i}}^{\prime }+t$, where *t* denotes the translation matrix and *R* denotes the rotation matrix. Then the error term for the *i* pair of points is defined as $e_{i}=P_{i}-\left(R{P_{i}}^{\prime }+t\right)$.

For each current plane observation, it is necessary to find the associated match in the global map and create a new map plane if the above conditions cannot be satisfied. Once the two planes are matched, the in-plane points are associated with the relevant attributes of the planes instead of looking up from the ORB feature matching, which significantly reduces the search and optimization time. We denote the set of matched two in-plane points as $P_{\Pi }^{c,L}$, where $P_{\pi }^{c}$ denotes the points in the current plane of the current frame and $P_{\pi }^{L}$ denotes the in-plane points as landmarks in the local map.

### 3.8. Position Estimation

**Figure 4:**
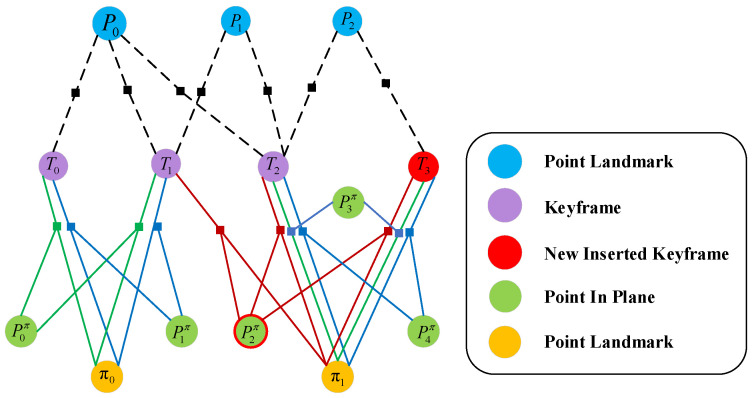
Factor graph optimization.

According to the previous section, we obtain the matched points, the matched planes, and the matched in-plane points. Therefore the tracked points, planar features, and in-plane points are then used to construct the cost function to estimate the poses jointly. In contrast, the g2o [38] graph optimization, a method for constructing error functions from edges and vertices, is often used to optimize the poses in the pose-optimization problem of SLAM. In g2o, binary edges (one edge and two vertices) are the most frequently used optimization method; therefore, based on these three constraints, a factor graph can be constructed as in Figure 4.

The optimization process of the non-planar in-plane points corresponds to the blue node in Figure 4. The cost function of the reprojection error of the non-planar map point $P_{w}^{c}$ in the world coordinate system of the current frame concerning the camera coordinate system is expressed as:

(11) $$E_{P}={∥P_{c}^{c}-p\left(T_{c,w}^{c}P_{w}^{c}\right)∥}_{\Omega }\;$$

where $P_{c}^{c}$ denotes the observation of the point in the camera coordinate system (subscript c) for the current frame (superscript *c*), p(.) denotes the projection of the map point in the global coordinate system in the camera coordinate system, and ${∥x∥}_{\Omega }$ is expressed based on the Marxian criterion and is equivalent to $X^{⊤}\Omega^{-1}X$, and Ω denotes the covariance matrix.

The process of plane optimization is equivalent to the orange node in Figure 4. Similarly, the cost function of the constructed out plane error for the current frame world plane $\pi_{w}{}^{c}$ transformed into camera plane $\pi_{c}^{c}=T_{c,w}^{-⊤}\cdot \pi_{w}{}^{c}$ is

(12) $$E_{\pi }={∥\log\left(Q{\left(T_{c,w}^{-⊤}\cdot \pi_{w}{}^{c}\right)}^{-1}⊗Q\left(\pi_{c}^{L}\right)\right)∥}_{\Omega }\;$$

where Q(•) is denoted as the normalized transfer function of a four-dimensional vector. As the green node in Figure 4, in-plane point $P^{\pi }\subset P_{\Pi }^{c,L}$, we find the corresponding in-plane point in the matching plane for reprojection to construct the cost function:

(13) $$E_{p^{\pi }}={∥P_{\pi }^{c}-p\left(T_{cw}P_{\pi }^{L}\right)∥}_{\Omega }$$

In summary, the current pose can be optimized using a combination of points, planes, and in-plane points:

(14) $$T_{c,w}^{c}=\underset{T_{c,w}^{c}}{\arg\min}\left(\sum_{P^{c,L}}\rho \left(E_{p}\right)+\sum_{\Pi^{c,L}}\rho \left(E_{\pi }\right)+\sum_{P_{\Pi }^{c,L}}\rho \left(E_{p^{\pi }}\right)\right)$$

where ρ(.) is a Huber robust kernel function.

We extracted feature points and a sufficient number of in-plane points based on CAPE in the depth map and the advantage of the cytoplasmic center of mass brought about by region-based growth. In the case of successful tracking of the previous frame, the homogeneous model is commonly used to track and predict the locus pose of the current frame. The construction of cost functions for points, planes, and in-plane points with planar correlation coefficients is completed based on local point landmarks or a global search for planar landmarks. Each local map contains several keyframes; the current frame shares point and plane landmarks with the keyframes, and the points in the same plane are indirectly linked together by sharing plane correlation coefficients. Thus, optimizing the above conditions of the completed local map makes the current locus more accurate.

## 4. Experimental Design and Analysis

To evaluate the performance of YPD-SLAM, we conduct relevant experiments on the dynamic public scene TUM [39] (RGB-D) dataset. All experiments are done on an Intel Core i5-12400F desktop computer with 16 GB RAM and Ubuntu 18.04 system without GPU acceleration.

The TUM RGB-D dataset was published by the Computer Vision Lab at the Technical University of Munich and consists of 39 sequences recorded by Kinect sensors in different indoor scenes. This paper used sequences under the category “Dynamic Objects” for evaluation tests. The sequences can be divided into two categories; one is a low dynamic sequence called “Sitting” fr3/sitting (fr3/s for short): fr3/s_xyz, fr3/s_half, and fr3/s_xyz, which describes two people sitting in front of a table and talking while doing actions; the other category is the highly dynamic sequence fr3/walking (fr3/w for short): fr3/w_half, fr3/w_rpy, fr3/w_static and fr3/w_xyz. This sequence describes two people moving in the background foreground and exchanging positions. Highly dynamic sequences in which dynamic objects occupy a large portion of the field of view severely affect the positional estimation and are very challenging for SLAM systems.

In the half-sphere (half for short) sequence, the camera moves along the hemisphere; in the rpy sequence, the camera performs panning and pitching motion; in the static sequence, the camera’s position remains constant; and in the xyz trajectory, the camera moves along the axis, axis and axis, respectively.

First, we qualitatively evaluate and analyze the dynamic object detection and planar detection effects of YPD-SLAM. Then, the system is quantitatively compared and analyzed with state-of-the-art dynamic SLAM methods and planar-based SLAM methods, and the enhancement of YPD-SLAM in dynamic scenes is quantified. Finally and most importantly, we evaluate the system in real-time, calculate the running time of each segment, and compare the same with the running time of the state-of-the-art algorithms mentioned above.

### 4.1. Dynamic Point Rejection

**Figure 5:**
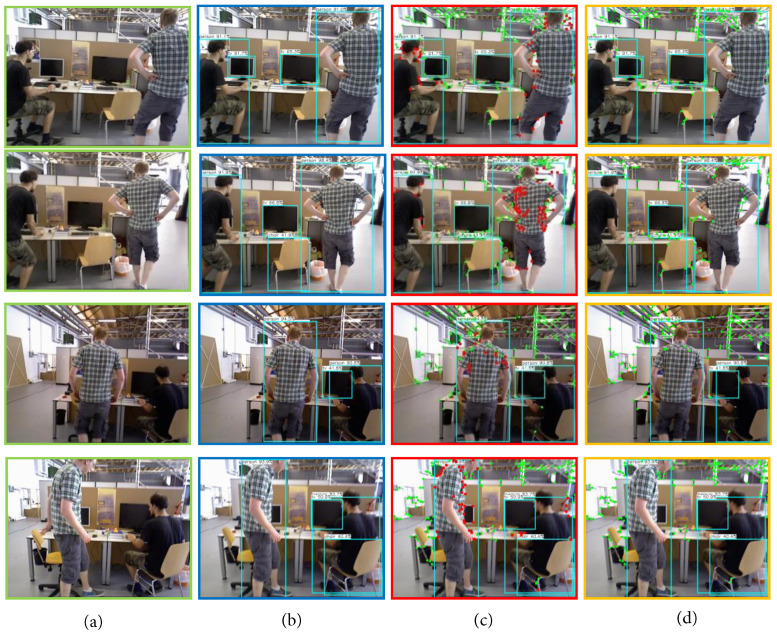
Dynamic point rejection, from left to right: (**a**) original image; (**b**) target detection; (**c**) dynamic feature point detection for dynamic objects; (**d**) rejection of dynamic feature points.

First, the dynamic point rejection section is shown in TUM at fr3/w_xyz. As shown in Figure 5, from left to right, (a) the original image; (b) the target detection using Yolo-FastestV2; (c) the dynamic and static points filtered out by dynamic point checking under the premise of target detection; (d) after removing the dynamic points. From (a) to (b), we can find that several classes of objects in the visual field trained with the COCO dataset have been detected, including “person”, “tv”, and “chair”. Subfigure (c) shows that only the highly dynamic object “person” is detected, and dynamic point detection is performed in the corresponding “Bounding Box”. Compared with the traditional SLAM algorithm that removes all the feature points from the target detection box, this paper retains the static points to remove the relative dynamic points, which increases the number of feature points that can be used for pose estimation. Subfigure (d) shows the final figure after removing the dynamic points. It can be seen that the method in this paper is effective in removing the effect of dynamic points.

### 4.2. Plane Detection

Following the above, three sequences of fr3/w_xyz, fr3/w_rpy, and fr3/w_half are selected to demonstrate the cases where the number of detectable feature points is small and the low-texture scenes are many or even the tracking is lost.

**Figure 6:**
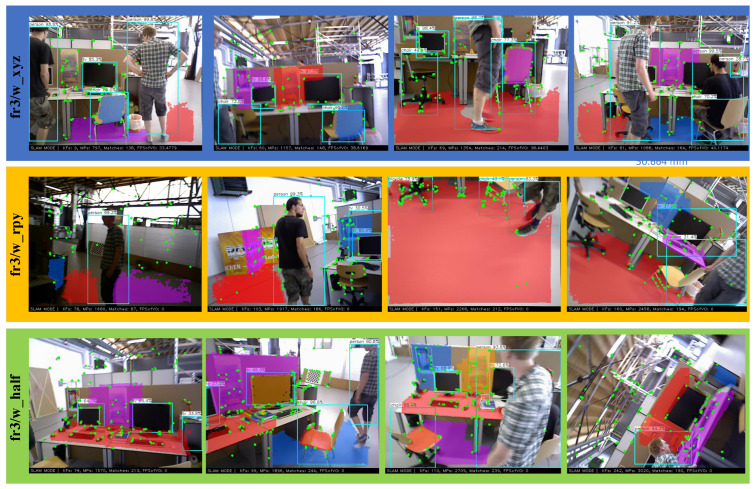
Planar detection, from top to bottom, for the highly dynamic sequences fr3/w_xyz, fr3/w_rpy, and fr3/w_half.

As shown in Figure 6, the third image of fr3/w_xy, fr3/w_rpy, and the second and fourth images of fr3/w_half all offer a large number of planes with very few feature points on them. The method in this paper can cleverly solve the following problem—adding planar detection when the number of feature points is insufficient, and the number of points available for pose estimation becomes even less due to dynamic point rejection. The center of mass of the cell is back-projected to the point cloud plane after the planar region is grown. This allows the 3D points in the map to be recovered based on the camera model, significantly increasing the number of observable points.

**Figure 7:**
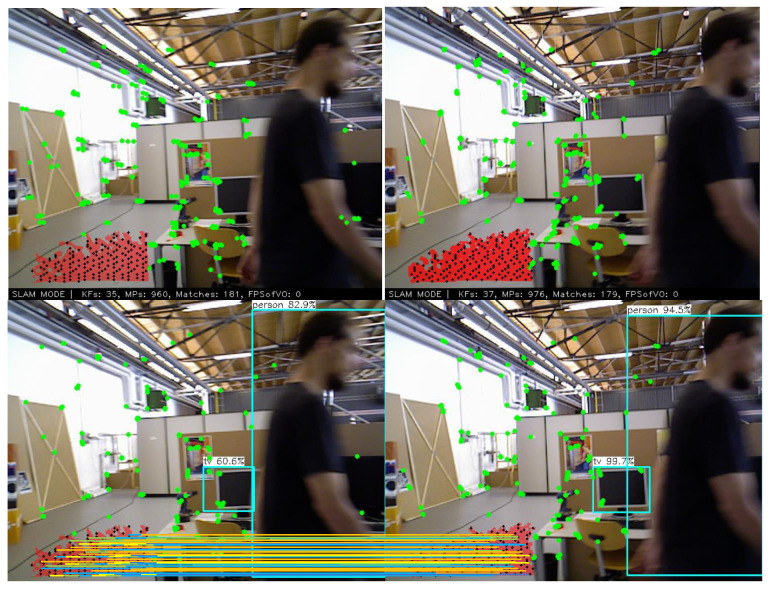
Points in the 3D plane that have been matched. Their matching relationship is expressed in RGB images in the form of two-dimensional coordinate lines.

As shown in Figure 7, due to the low confidence of the edge points in the plane, we skip the set of points closest to the edge in the ICP process. The matching corresponding 3D points are back-projected to the camera plane, and the matching corresponding points are added to the line segment description in the RGB map.

**Figure 8:**
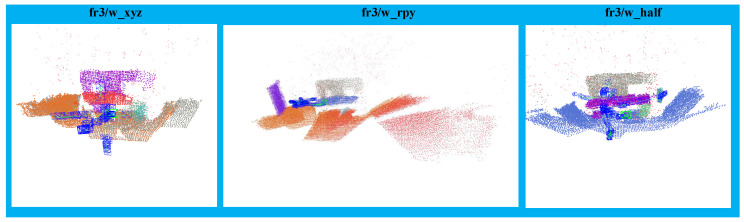
Global map.

As seen in Figure 8, planes and points within planes appear successfully and realistically on the global map.

### 4.3. Positioning Accuracy

For the convenience of expression, we use this simplified expression for the following parts: YPD (YPD-SLAM); ORB2 (ORB-SLAM2); SP-SLAM (SP); DS (DS-SLAM); RDMO (RDMO-SLAM); RDS (RDS-SLAM).

In this paper, absolute trajectory error (ATE) and relative attitude error (RPE) [39] are used to evaluate the positioning accuracy of the algorithms. The Root Means Square Error (RMSE) is selected as the evaluation metric for quantitative comparison with several state-of-the-art algorithms. In this paper, the most classic original algorithm ORB-SLAM2, plane-based SP-SLAM, and semantic-based DS-SLAM, RDMO-SLAM, and RDS are selected for comparison.

As in Table 1 for the low dynamic sequence fr3/s_ static, the RMSE of YPD is improved by only 11.30%. The original algorithm ORB2 shows strong robustness, whereas RDS performs the best. This is because RDS can handle low dynamic sequences well and has achieved a high level of localization accuracy, so the improvement to YPD is limited. YPD significantly improved for high dynamic sequences over the original ORB2, especially in the fr3/w_xyz sequence, where the RMSE improvement reaches 98.21%. Since the fr3/w_ rpy dataset undergoes a significant angle rotation, the target detection network YolofatestV2 less effective than the semantic segmentation network Segnet. However, the compensation by plane and in-plane points makes the difference of RMSE between YPD and RDS for ATE under fr3/w_ rpy dataset tiny. In order to meet the fast operation of the system we lost some of the accuracy within the acceptable range. Furthermore, except RDS, YPD still outperforms several other algorithms. YPD in fr3/w_half and fr3/w_static were each improved by 93.90% and 86.87%, respectively, compared to ORB2. According to the comparison with the other SLAM algorithms mentioned above in Table 1, it is found that the RMSE of YPD is the smallest in fr3/w_static and fr3/w_xyz, which are marked in bold in the table. In general, YPD and state-of-the-art RDS systems almost achieve similar position accuracy in ATE; however, the running time is much less than in RDS, see Section 4.4.

According to the data in Table 2 and Table 3, it can be obtained that the trends of a relative translation error and relative rotation error in the TUM dataset are similar to those of ATE. The overall performance is that the high dynamic sequences have a significant enhancement effect, and the low dynamic sequences have a minor enhancement.

To further highlight our contribution, we set up a set of experiments for quantitatively evaluating the contribution of each to the system performance based on the RMSE of ATE. We use the abbreviation YFV2 to denote YoloFatestV2 for easy tabular presentation. Only “ours(4)” represents YPD-SLAM, while “ours(1), ours(2), and ours(3)” represent only partial components and not the complete YPD-SLAM.

As shown in the Table 4, comparing ours(1), ours(2), and ours(4), only ours(4) shows the best performance in face of both dynamic and low-texture scenes, although all have improved. It is worth noting that, in fr3/w_rpy, where low-texture scenes occur more often, ours(1) and ours(2) do not perform very well, with ATE improvements of only 40% and 22.8%, respectively. However, combining them as “ours(4)”, the ATE improves by 96.39% in RMSE. Observing ours(3) and ours(4), the RMSE of ATE decreases after adding in-plane points constraint to the system.

**Figure 9:**
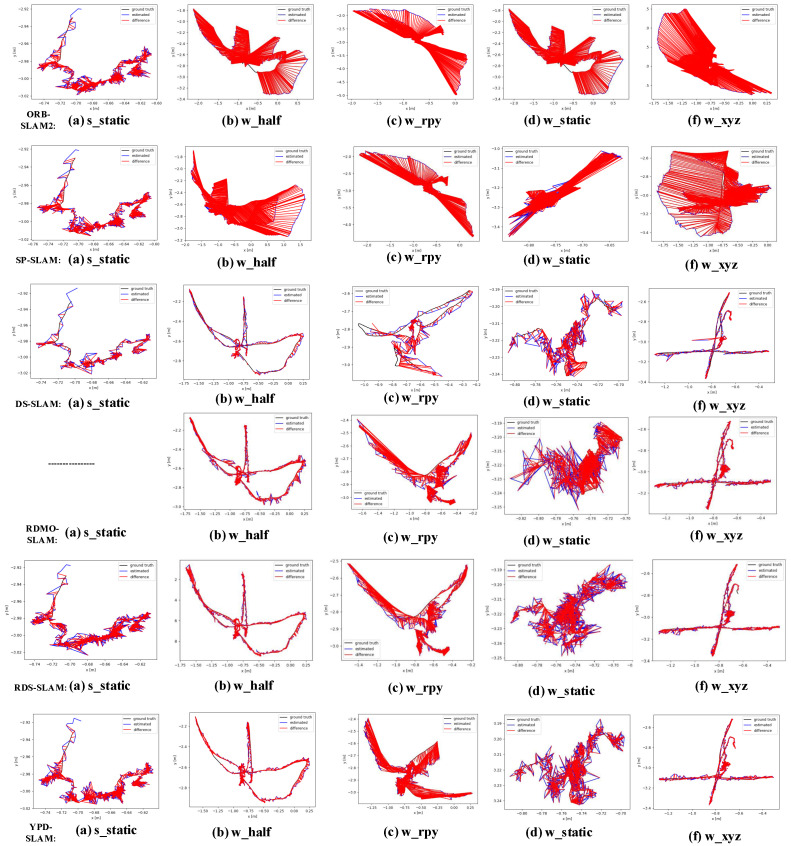
Error plot for ATE. Black represents the groundtruth, blue represents the estimated trajectory, and red represents the gap between the estimated trajectory and the real trajectory.

In addition to the tables, we have drawn figure forms that are easy to observe. Figure 9 compares the estimated and absolute trajectories of YPD with ORB, SP, DS, RDMO, and RDS for low and high dynamic sequences and the error plots. The red line indicates the error between the estimated and true trajectory.

**Figure 10:**
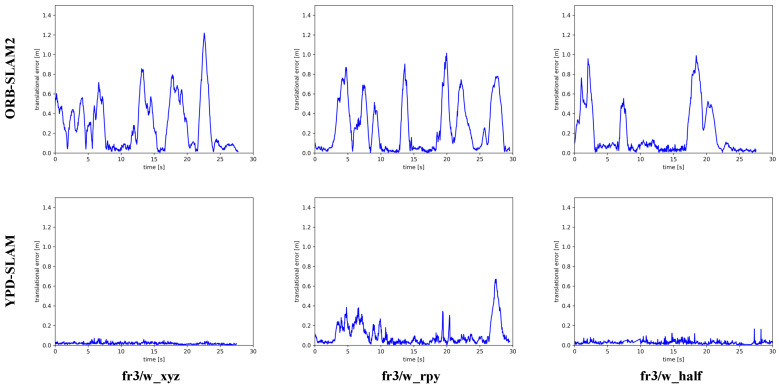
The RPE results of ORB-SLAM2 and YPD-SLAM in fr3/w_xyz, w_rpy, and w_half.

Figure 10 compares the RPE plots of the ORB and the YPD. Both figures clearly show that the ATE and RPE of the YPD are at a relatively low level and are generally better than the ORB and SP. Again, this indicates that the system achieves similar tracking performance to the advanced DS and RDMO. However, the DS and RDMO perform poorly in terms of real-time performance.

### 4.4. Real-Time Performance

The time cost is an essential indicator of system quality in practical applications, and this paper also focuses on real-time performance in indoor environments. As shown in Table 5, it can be seen from the previous article that YOLO-FastestV2 using the Shufflenet V2 backbone network, runs extremely fast, and the target detection thread runs in parallel with the tracking thread in the whole system.The average running time of the target detection module is only 4.40 ms per frame when tested under five sequences of TUM, and the average time spent for CAPE plane extraction is only 3.73 ms per frame. Since the tracking thread sets the requirement to wait for the result of the target detection thread, we merge the time of target detection into the tracking thread time. The average time for the whole tracking thread to run is only 42.68 ms, which fully satisfies the real-time requirement. Finally, the average local optimization time is only 53.83 ms after adding the in-plane and in-plane point constraints.

Table 6 shows the execution time of YPD-SLAM compared with ORB-SLAM2, SP-SLAM, DS-SLAM, RDS-SLAM, and RDMO-SLAM for the leading models in the tracking thread for the TUM dataset. Although the overall time consumption of the SP-SLAM tracking thread is less, its local BA optimization for the assumed plane is very time-consuming, up to 141.1678 ms. In terms of the speed of processing semantic information, the model segmentation of DS takes 37.57 ms, while the target detection of YPD takes only 4.4 ms, presenting an improvement of 88.29%. Unlike DS, only the points inside the Lable frame are checked and culled in this paper, which can significantly reduce the time consumed by the global dynamic point screening. Therefore, the Dynamic Points Culling section takes 16.08 ms, which is 45.51% better than the Move Consistency Check section of DS. Therefore, the proposed system fully meets the real-time requirement.

In order to select the case with more keyframes for a fairer evaluation, the table selects the runtime of RDS and RDMO at 15HZ. The RDMO combined with Mask R-CNN semantic segmentation phase takes about 205.42 ms, and the RDS combined with Segnet segmentation takes about 35.04 ms. According to the table, we know that the target detection of YPD-SLAM takes only 4.4 ms. Since RDMO and RDS add semantic threads on keyframes and optimize them, this brings the benefit of significantly reducing the system tracking time, but they are still GPU-based, and it is difficult to cope with fast motion and motion blur.

Under COCO data, the network model size of Mask-RCNN is 245.6 MB, and that of Segnet is 29 MB, and both require GPU-accelerated inference, which cannot be done on the CPU. Therefore, RDS/RDMO is less lightweight and requires a higher hardware platform. In contrast, the network model of YoloFastestV2 is only 1.3 MB, which is more efficient and dramatically reduces the hardware requirements.

In the TUM dataset, the tracking time per frame for RDMO/RDS is 50–65 ms, while the YPD tracking time is only 42.68 ms. The tracking time is still improved by 14.6–34.33% without GPU. Furthermore, the presence of in-plane and in-plane points makes the global map more structured and realistic. This will increase the 3D perception of the system in the real environment. YPD-SLAM has a higher value for wide engineering deployment and applications considering cost, time, and accuracy.

In addition, planes and in-plane points make the global map more structured and realistic. This will increase the system’s 3D perception of the real environment. YPD-SLAM has a higher value for various engineering deployments and applications, considering the cost, time, and accuracy factors.

## 5. Conclusions

In this paper, we propose a real-time VSLAM system based on CAPE plane extraction and YPD-SLAM based on YOLO-FastestV2 target detection for indoor dynamic environments, which mainly adds semantic threads to ORB-SLAM2 and adds planes and in-plane points to the original tracking threads and adds a back-end optimization process.

To overcome the problem that traditional SLAM pose estimation is vulnerable to dynamic objects, pixel-level segmentation networks (e.g., Mask R-CNN) are very time-consuming. This paper uses the fastest and moderately accurate target detection network, YOLO-FastestV2, which makes the semantic thread execution speed significantly faster. It also combines the optical flow method and dyadic geometry to reject dynamic feature points of highly dynamic targets between consecutive frames. In the experimental session, the rejection effectively meets the real-time requirements (the average time spent per frame for target detection and dynamic point rejection is 20.48 ms). The number of feature points used for pose estimation is insufficient, and the number of indoor low-texture scenes is high after rejection. We recover the map by CAPE planar extraction and depth map back-projection to the point cloud, increasing the planar constraint of indoor structures and in-plane point constraint. Matching points, planar landmarks, and in-plane points of the global map jointly optimize the poses, dramatically improving the system’s localization accuracy and robustness.

The experimental results based on the TUM dataset show that YPD-SLAM has excellent robustness, accuracy, and real-time performance in a dynamic indoor environment. Yolo-FastestV2 target detection network and morphological CAPE plane extraction are both highly versatile. The whole system can be performed on CPU, which dramatically reduces the hardware cost and is advantageous for deployment on various platforms.

However, YPD-SLAM still has some shortcomings. For example, a large rotation causes the target detection network abnormal, leading to false identification, rejection, and even fewer tracking failures in the experiments. Therefore, more object constraints and feature point selection methods must be added. To achieve the effect of real-time, the plane extraction in this paper has a lot of edge jaggedness due to erosion expansion operation will affect the plane effect. However, in reality, this jaggedness does not exist. This work may focus on extending the morphological plane extraction to better plane extraction networks in the future.

## Figures and Tables

**Table 1:** Experimental RMSE results of absolute trajectory error (ATE). Our method is YPD-SLAM, and Improvement stands for comparison with the original algorithm ORB-SLAM2.

Sequence	Original	Plane-Based	Semantic-Based				Ours	Improvement
	ORB-SLAM2	SP-SLAM	DS-SLAM	RDMO-SLAM	Paper [24]	RDS-SLAM	YPD-SLAM	
fr3/s_ stati	0.0087	0.0090	0.0065	0.0066	0.0058	**0.0039**	0.0077	11.30%
fr3/w_half	0.4811	0.5364	0.0303	0.0304	-	**0.0291**	0.0294	93.90%
fr3/w_rpy	0.9548	0.8324	0.4442	0.1283	0.0612	**0.0128**	0.0345	96.39%
fr3/w_static	0.0476	0.2745	0.0081	0.0126	0.0069	0.0215	**0.0072**	86.87%
fr3/w_xyz	0.9026	0.5927	0.0247	0.0226	0.0565	0.0565	**0.0161**	98.21%

**Table 2:** Experimental RMSE results of translational relative pose error (RPE). Our method is YPD-SLAM, and Improvement stands for comparison with the original algorithm ORB-SLAM2.

Sequence	Original	Plane-Based	Semantic-Based			Ours	Improvement
	ORB-SLAM2	SP-SLAM	DS-SLAM	RDMO-SLAM	RDS-SLAM	YPD-SLAM	
fr3/s_static	0.0106	0.0096	**0.0078**	0.0090	0.0050	0.0093	12.15%
fr3/w_half	0.3050	0.3008	0.0297	**0.0294**	0.0295	0.0318	89.57%
fr3/w_rpy	0.3767	0.3921	0.1503	0.1396	**0.0245**	0.0504	86.63%
fr3/w_static	0.0689	0.1665	0.0102	0.0160	0.0221	**0.0094**	86.39%
fr3/w_xyz	0.3945	0.3990	0.3330	0.0299	0.0269	**0.0110**	97.21%

**Table 3:** Experimental RMSE results of rotational relative pose error (RPE). Our method is YPD-SLAM, and Improvement stands for comparison with the original algorithm ORB-SLAM2.

Sequence	Original	Plane-Based	Semantic-Based			Ours	Improvement
	ORB-SLAM2	SP-SLAM	DS-SLAM	RDMO-SLAM	RDS-SLAM	YPD-SLAM	
fr3/s_static	0.3004	0.2943	0.2735	0.3233	**0.1520**	0.2732	9.06%
fr3/w_half	6.0318	5.9273	0.8142	0.7915	**0.7332**	0.8648	85.66%
fr3/w_rpy	7.2879	7.5682	3.0042	2.5472	**0.4973**	1.1675	83.98%
fr3/w_static	1.2309	2.8911	0.2690	0.3385	0.4944	**0.2508**	79.63%
fr3/w_xyz	7.5193	7.6226	0.8266	0.7990	0.7768	**0.6535**	91.31%

**Table 4:** For the TUM dataset, different algorithms are composed to obtain the RMSE of ATE.

Sequence	Original	YPD-SLAM			
	ORB-SLAM2	Ours(1) Yolo-FatestV2	Ours(2) Plane + In-Plane Points	Ours(3) YFV2 + Plane	Ours(4) YFV2 + Plane + In-Plane Points
fr3/s_ static	0.0087	0.0089	0.0091	0.0078	**0.0077**
fr3/w_half	0.4811	0.0427	0.0526	0.0313	**0.0294**
fr3/w_rpy	0.9548	0.5738	0.7372	0.0763	**0.0345**
fr3/w_static	0.0476	0.0158	0.2743	0.0102	**0.0062**
fr3/w_xyz	0.9026	0.0352	0.5731	0.0467	**0.0161**

**Table 5:** Average operating time of principal components (ms).

Main Components		ORB Feature Extraction	Object Detection	Dynamic Points Culling	Plane and Points in-Plane Extraction	Matching and Landmarks Tracking	Total	Local Optimization
	Thread	Tracking	Object Detection	Tracking	Tracking	Tracking	-	Local Mapping
Seq								
fr3/s_ static		9.56	4.14	16.84	3.62	4.38	38.54	28.66
fr3/w_half		9.80	4.60	15.05	3.82	11.66	44.92	79.75
fr3/w_rpy		10.43	4.57	15.31	3.93	11.97	46.21	51.78
fr3/w_static		10.07	4.11	17.15	3.52	5.58	40.42	54.37
fr3/w_xyz		11.43	4.59	16.05	3.76	7.45	43.27	54.58
Average		10.26	4.40	16.08	3.73	8.21	42.68	53.83

**Table 6:** The average operation time of principal components in various latest algorithms (ms).

Systems	Hardware Platform	Main Module In Tracking Thread					Total
ORB-SLAM2	Intel Corei5-4288U CPU	Feature Extraction	Initial Pose Estimation	Track Local Map	-	-	
	-	21.6900	3.1500	11.5300	-	-	36.37
SP-SLAM	Intel Core i7	Feature Extraction	Plane Segmentation	Supposed Plane Generation	Matching and Tracking Landmarks	-	
	-	11.55	6.8	7.06	11.88	-	37.29
DS-SLAM	Intel Core i7	Feature Extraction	Move Consistency Check	Semantic Segmentation	-	-	
	P4000 GPU	9.38	29.51	37.57			76.46
RDS-SLAM	-	Segmentation and mask generation	Update moving probability	Semantic-based optimization	-	-	
	GeForce RTX 2080Ti GPU	205.42	0.17	0.54	-	-	50–65
RDMO- SLAM	-	Segmentation and mask generation	Optical flow	Update moving probability	Velocity Estimation and Label Prediction	-	
	GeForce RTX 2080Ti GPU	35.04	54	0.14	4.1	-	50–65
YPD-SLAM	Intel Corei5-12400F CPU	Feature Extraction	Object Detection	Dynamic Points Culling	Plane and Points in-Plane Extraction	Matching and landmarks tracking	
	-	10.2600	4.4000	16.0800	3.7300	8.2100	42.68

## Data Availability

The datasets used in this paper are the publicly available COCO dataset and the publicly TUM dataset. They can be downloaded at the following links: 1. TUM RGBD-dataset (https://vision.in.tum.de/, accessed on 20 August 2022); 2. COCO 2017dataset (https://cocodataset.org/, accessed on 20 August 2022).
